# Sleep quality and mindfulness: mediating the relationship between neuroticism and subjective well-being in early adolescents

**DOI:** 10.1007/s12519-025-00979-3

**Published:** 2025-10-29

**Authors:** Xiaopeng Ji, Xun Fang, Patrick W. L. Leung, Jianghong Liu

**Affiliations:** 1 School of Nursing, College of Health Sciences, University of Delaware, Newark, DE USA; 2 Department of Psychology, The Chinese University of Hong Kong, 3/F, Sino Building, Shatin, Hong Kong China; 3 Department of Family and Community Health, University of Pennsylvania School of Nursing, 418 Curie Blvd., Room 424, Claire M. Fagin Hall, Philadelphia, PA USA

**Keywords:** Adolescents, Chronotype, Mediation, Mindfulness, Neuroticism, Sleep, Subjective well-being

## Abstract

**Background:**

Good sleep quality, appropriate sleep timing, and mindfulness support emotional and mental health well-being. However, few studies have examined their role in the association between neuroticism and subjective well-being (SWB) among adolescents. This study investigated their potential moderating and mediating effects on the neuroticism–SWB relationship in early adolescents.

**Methods:**

We enrolled 1110 adolescents in the China Jintan Child Cohort—Wave II study, with 543 providing complete data on sleep, neuroticism, and SWB (2011–2013, 12.98 ± 0.88 years old, 49% females) and 188 providing trait mindfulness data (2013–2014). The sleep variables included sleep quality (Pittsburgh sleep quality index) and chronotype (mid-sleep time on weekends corrected for sleep debt). SWB was measured via the Oxford happiness questionnaire, neuroticism was measured via the Big Five Inventory, and mindfulness was measured via the five facet mindfulness questionnaire. Linear regression was used to estimate the moderating effect, and generalized structural equation modeling was used to examine the mediating effects.

**Results:**

Neuroticism (*b* = −5.07, *P* < 0.001) significantly predicted lower SWB, which was mediated by poor sleep quality (*β* = −3.76, *P* = 0.002) and trait mindfulness (*β* = −2.13, *P* = 0.002). No moderating effects were found for sleep quality or mindfulness (*P* > 0.05). Chronotype was not a moderator or a mediator between neuroticism and SWB, although a moderate (vs. late) chronotype was independently associated with better SWB (*b* = 5.91, *P* = 0.04).

**Conclusions:**

Poor sleep and mindfulness mediate, but do not moderate, the relationship between neuroticism and SWB. While the late chronotype predicts poorer SWB, it does not contribute to the neuroticism–SWB relationship. The findings underscore the importance of healthy sleep and mindfulness-based strategies to support well-being in early adolescents high in neuroticism.

**Graphical abstract:**

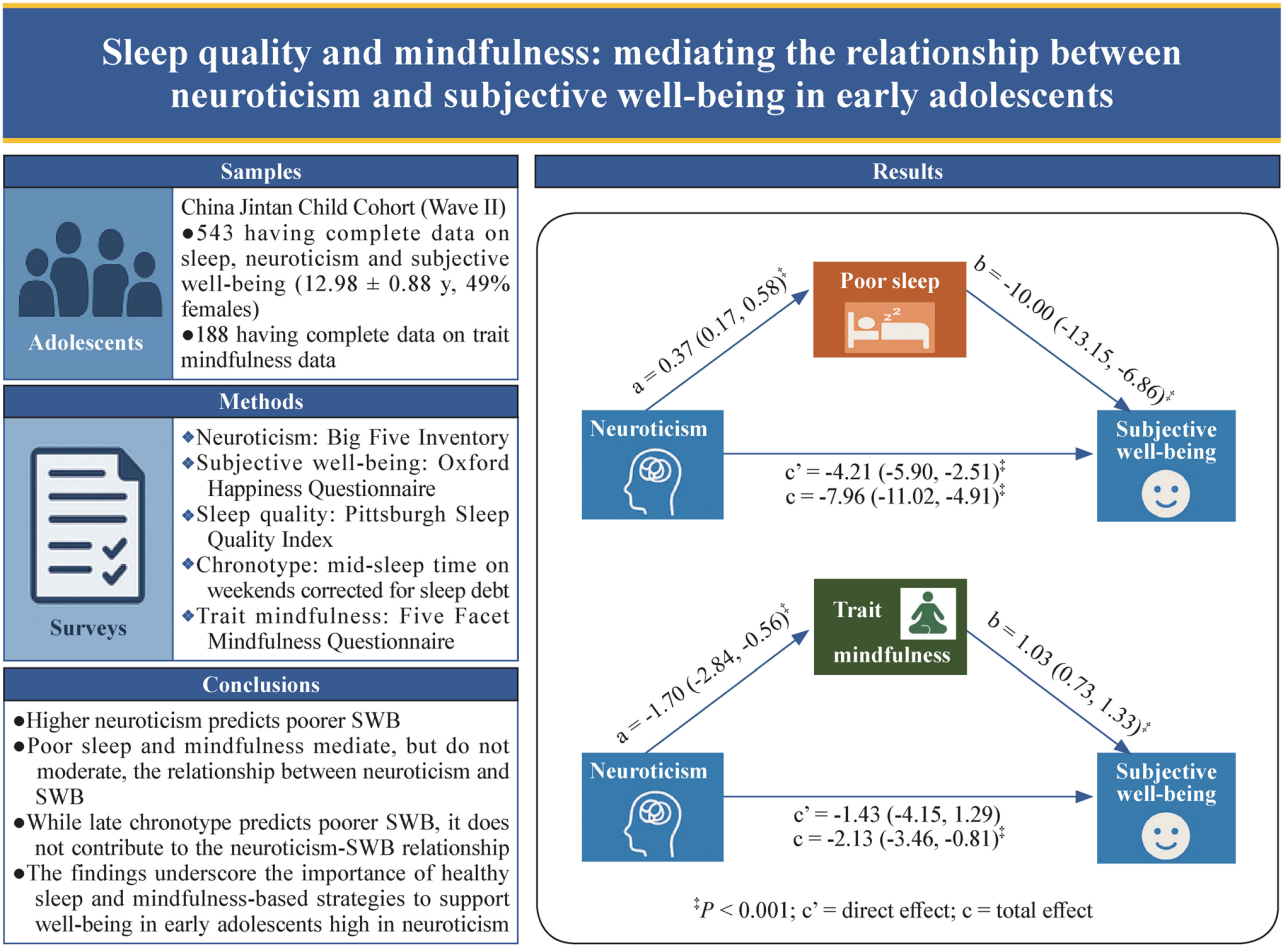

## Introduction

Sleep deprivation (< 8 hours), poor sleep quality (e.g., difficulty falling asleep and sleep fragmentation) [1–6], and a delayed circadian phase [7] (e.g., later sleep and wake times) are public health issues in adolescents. Up to 75% of adolescents report any type of sleep concern [1–7]. Poor sleep health has been negatively associated with subjective well-being (SWB) [8–10], as measured by a cognitive evaluation of life as a whole (e.g., life satisfaction) or a subjective process related to positive affect (e.g., happiness) [11]. Specifically, short and poor sleep are related to low SWB levels both concurrently and over time [8–10]. SWB further contributes to mental health, healthy behaviors, and academic performance in adolescents [12–14]. These challenges may be more pronounced in Asian adolescents, where delayed melatonin onset during puberty [15] intersects with academic pressures and cultural expectations, increasing the risk of poor sleep compared with peers in Western countries [2].

Mindfulness is another modifiable factor related to SWB. Mindfulness can be considered a personality trait (dispositional mindfulness), representing the innate capacity of paying and maintaining attention to present-moment experiences with an open and non-judgmental attitude or a state of present-moment awareness that is subject to change [16, 17]. High levels of mindfulness, at both the trait and state levels, have been found to be associated with better SWB in adults [18].

Neuroticism, a negative personality trait, also contributes to decreased SWB. Neuroticism is characterized by a tendency to experience psychological distress and negative emotions [19]. Neuroticism tends to peak during adolescence and then generally declines or stabilizes into adulthood [20]. Owing to hyperreactivity to negative environmental stimulation [21], adolescents high in neuroticism often perceive stress events as more threatening [22] and thus are more susceptible to emotional regulation difficulties and behavioral problems [23]. Individuals high in neuroticism tend to report poor SWB [18, 24]. Genome-wide analyses suggest that loci regulating expression in the central nervous system and adrenal/pancreatic tissues are correlated with both neuroticism and SWB [25].

Both sleep health and mindfulness may influence the relationship between neuroticism and SWB, offering potential targets for interventions to disrupt the vicious cycle. In studies focused primarily on adults, neuroticism is associated with worse sleep quality, difficulty falling asleep, increased daytime sleepiness, and a reduced tendency toward a morningness chronotype [26–30], possibly mediated through metacognitive, cognitive, and emotional factors [27–30]. Among older adults, perceived sleep quality mediates the association between neuroticism and quality of life [31], and better sleep quality reduces the strength of the relationship between high neuroticism and poor frontal executive function [32]. Moreover, there is an interaction effect between neuroticism and chronotype on depression. With lower neuroticism, the association between the eveningness chronotype and depressive symptoms in young adults becomes less pronounced [33]. In terms of mindfulness, meta-analyses have shown a strong, negative relationship between neuroticism and trait mindfulness [16, 17]. Mindfulness has also been found to partially mediate the associations between neuroticism and SWB in adults [18]. Additionally, prior research has shown that trait mindfulness can moderate the pernicious correlates of neuroticism, such as trait anger and depressive symptoms, with stronger associations shown in young adults low in mindfulness [34].

Despite accumulating evidence supporting the interplay between sleep, mindfulness, neuroticism, and SWB, limited research has investigated the potential moderating or mediating mechanisms of sleep quality and mindfulness in the neuroticism–SWB pathway during adolescence [35]. This is important for two reasons: first, coincident with an increase in neuroticism during adolescence [20], there is a progressive decline in sleep health [6] and SWB [35] during this developmental period. Thus, adolescents are at particularly high risk for downstream health outcomes. Second, neuroticism, which is generally stable across the lifespan, is less likely to be modified [36, 37]. A better understanding of the malleable variables that are related to the relationship between neuroticism and SWB, such as sleep and mindfulness, will inform interventions for children and adolescents.

The present study draws upon Drs. Brown and Barlow’s dimensional classification framework [38] and the diathesis-stress model [39]. Neuroticism represents a higher-order vulnerability factor that confers increased risk for psychological distress and compromised well-being, particularly during early adolescence, when biological, social, and academic stressors intensify. Consistent with the diathesis-stress model, neuroticism functions as a predisposing vulnerability that interacts with proximal life stressors (e.g., academic pressures, peer conflicts) to precipitate maladaptive outcomes through disruptions in self-regulatory systems, including sleep‒wake cycles and attention regulation processes underlying mindfulness. From this perspective, poor sleep quality and diminished mindfulness capacity may represent both consequences of neurotic vulnerability and additional risk factors that compound the pathway from personality predisposition to reduced SWB.

Therefore, in this cross-sectional study of 543 adolescents (12.98 ± 0.88 years old), we aimed to investigate two research questions: first, do sleep, circadian preferences and mindfulness moderate the association between increased neuroticism and decreased SWB, and second, do sleep, circadian preferences and mindfulness mediate the association between neuroticism and SWB among adolescents? Poor sleep health was operationalized as overall sleep impairment indicated by multiple sleep domains, such as sleep duration, sleep quality, sleep disturbances, and daytime sleepiness [40]. Circadian preferences were operationalized as early (bedtime before 2 a.m.), moderate, and late chronotypes [41, 42]. SWB was operationalized as positive affect, indicated by perceived happiness. Given that neuroticism is poorly susceptible to change, knowledge about modifiable factors that are connected to the association between neuroticism and SWB has significant implications for health promotion during adolescence and beyond.

## Methods

### Participants and procedures

This study is part of the China Jintan Child Cohort study, which is aimed at investigating the impact of environmental exposure on neurobehavioral development [43, 44]. Using a multi-stage sampling approach, the study team initially recruited 1656 preschoolers (55.5% males) who represented children from urban, suburban and rural school districts in Jintan, China, in 2004. Jintan is a small county-level city in Jiangsu Province, China. The participants were classified into lower, middle, and upper cohorts according to their preschool year. The study team followed up on multiple health aspects when participants were at preschool in 2004–2007 (Wave 1, 3–5 years old), in their last month of sixth grade in 2011–2013 (Wave II, 11–14 years old), and in middle school in 2013–2018 (Wave III, 13–17 years old) [43, 44]. The detailed sampling and research procedures of this larger cohort study have been described elsewhere [43, 44].

This cross-sectional study included 543 adolescents (out of 1110) who had complete data on sleep, neuroticism, and SWB at Wave II (11–14 years old) to examine the role of sleep health in the relationship between neuroticism and SWB. Of these, 188 adolescents who also provided data on trait mindfulness collected from 2013 to 2014 were included in analyses of the role of mindfulness. Data were not missing at random. Compared with those without mindfulness data, participants who had complete data on mindfulness were one year older (*t* = −16.26, *P* < 0.001) and had a higher rate of poor sleep (40% vs. 31%, *χ*^2^ = 5.44, *P* = 0.02). To minimize the influence of sample differences, we accounted for age in the data analyses. Chi-square tests and *t* tests revealed no differences in key demographic and social factors [e.g., gender, residence district, socioeconomic status (SES)], neuroticism, SWB, or chronotype between those with (*n* = 188) and without (*n* = 355) mindfulness data.

## Measures

Adolescents self-reported their demographic information, as well as their SWB, sleep health, and neuroticism, via established questionnaires. The parents reported social and economic status variables, such as parents’ education and income.

### Subjective well-being

We measured SWB via the Chinese version of the Oxford happiness questionnaire (OHQ), which was originally designed by Hills and Argyle [45]. The QHQ is a compact scale that assesses subdomains, including positive cognition, positive cognition, social commitment, positive affect, sense of control, physical fitness, self-satisfaction, and mental alertness [45, 46]. It consists of 29 questions rated on a 6-point Likert scale ranging from 1 (strongly disagree) to 6 (strongly agree). The total score ranges from 29 to 174, with higher scores indicating greater happiness. The English version of the OHQ has shown good psychometric properties, with a Cronbach's *α* of 0.91 [45]. The Chinese version has demonstrated good reliability in populations such as university student samples [46], with a reported Cronbach’s *α* of 0.92. In our sample, the internal consistency was also good (Cronbach’s *α* = 0.86).

### Sleep health

#### Sleep quality

Adolescents completed the Chinese version of the Pittsburgh sleep quality index (PSQI), which assesses sleep quantity and quality over the previous month [40]. The PSQI is composed of 19 items that are grouped into seven components: sleep duration, self-rated sleep quality, sleep latency, sleep efficiency, daytime sleepiness and dysfunction, use of sleeping medication, and sleep disturbances. Each domain is scored from 0 to 3. Sleep duration was calculated on the basis of reported bedtime and wake time and categorized into four levels according to the adolescent sleep duration cutoff from the National Sleep Foundation [47, 48]: “ > 8 hours = 0, 7–8 hours = 1, 6–7 hours = 2, and < 6 hours = 3”. The component scores were summed to produce a global score ranging from 0 to 21, with total scores greater than 5 indicating poor sleep quality. The PSQI has demonstrated acceptable reliability and validity among Chinese adolescents, with an overall Cronbach’s *α* of 0.87 and subscale reliability coefficients ranging from 0.46 to 0.85. The cumulative variance explained by the principal components was 70.72% in Chinese adolescents [49].

#### Chronotype

Adolescents reported typical bedtime and waketime on weekdays (school days) and weekends (non-school days). We calculated the chronotype following the methodology established by Roenneberg et al. [47]. This approach uses the corrected mid-sleep time on free days (MSFsc), which accounts for accumulated sleep debt accrued during school days.

The mid-sleep time was the clock time halfway between bedtime and waketime [50]. When sleep duration on weekends was longer than that on weekdays, sleep debt was adjusted as follows: MSFsc = MSF – (average sleep duration on weekends-average weekly sleep duration)/2, expressed as the number of hours past night [7]. According to the healthy sleep midpoint (2–4 a.m.), we classified participants into early (before 2 a.m.), and moderate and late chronotypes (after 4 a.m.) [41, 42].

### Neuroticism

Adolescents completed the Chinese version of the Big Five Inventory (BFI-10), which measures neuroticism, extraversion, openness, conscientiousness, and agreeableness, with a 5-point Likert scale ranging from 1 (strongly disagree) to 5 (strongly agree) [51]. Scores on two questions from the neuroticism domain, “gets nervous easily” and “is relaxed, handles stress well” (reversed score), were averaged to reflect the neuroticism trait [52]. Higher scores suggest greater neuroticism. The BFI-10 has demonstrated acceptable test‒retest reliability and good evidence of both convergent and discriminant validity [52]. The Cronbach's *α* values for the neuroticism trait in the Chinese version of the BFI-10 range from 0.33 to 0.63 [51]. This variation in Cronbach’s alpha values reflects the trade-off between brevity and reliability often encountered in short personality measures used in community-based research.

### Trait mindfulness

The Chinese version of the five facet mindfulness questionnaire (FFMQ-SF) was used to measure adolescents' trait mindfulness [53]. The FFMQ-SF is a short form of the original 39-item measure [54], which measures the general tendency to be mindful. The FFMQ-SF consists of 24 questions assessing five mindfulness facets, including observing (e.g., I pay attention to sensations, such as the wind in my hair or sun on my face), acting with awareness (e.g., I am easily distracted; reverse-coded), non-judging of inner experience (e.g., I tell myself I shouldn’t be feeling the way I’m feeling; reverse-coded), non-reactivity to inner experience (e.g., I perceive my feelings and emotions without having to react to them), and describing (e.g., I can easily put my beliefs, opinions, and expectations into words). The respondents rated each item on a 5-point Likert scale from 1 (never or rarely true) to 5 (very often or always true). The scores of the 24 questions were averaged to form a composite score, with negatively worded items reverse-coded. Higher total scores indicate greater mindfulness. The FFMQ-SF has presented adequate construct and convergent validity as well as internal consistency [55], including for Chinese adolescents [56, 57].

### Covariates

Covariates included adolescents’ age at Wave II data collection, sex, and parents’ SES. Sociodemographic information was reported by the parents. SES status was calculated by summing the *Z* scores of four variables, the number of years of education, and the monthly wages of both the participant’s mother and father [58]. Higher SES scores represent better SES. The covariates were chosen on the basis of previous findings that they are important predictors of adolescents’ well-being and mental health [59, 60].

### Statistical analysis

Descriptive statistics were used to characterize continuous and categorical factors. At the bivariate level, Pearson correlations were used to test the interrelations among total scores of neuroticism, SWB, and mindfulness, and Student’s *t* test and ANOVA were used to test the differences in neuroticism and SWB scores between groups with different sleep qualities and chronotypes, respectively. We used a series of linear regression models (“regress” command in Stata) to estimate the relationships among SWB (dependent variable), neuroticism (independent variable), and sleep variables and mindfulness (moderators): (1) neuroticism was regressed onto the SWB scores, controlling for sex, age, SES composite score, and residence areas; (2) each moderator, including sleep quality, chronotype, and mindfulness, was added to the models separately; and (3) interaction terms between neuroticism and each moderator were then separately entered into the models to test the moderation effect. Linearity was assessed by plotting residuals against predicted values. The normality of the residuals was evaluated via histograms and Q‒Q plots. Visual inspection indicated an acceptable approximation.

Next, we estimated the direct and indirect effects of neuroticism on SWB via two models: a generalized structural equation model (GSEM, “gsem” command in Stata) with poor sleep quality as the mediator and an SEM (“sem” command in Stata) with mindfulness as the mediator. We first fitted a comprehensive model in which neuroticism (independent variable) influenced SWB (dependent variable), which was partially mediated by sleep quality. All covariates—sex, age, SES composite score, and residential location—were specified to have direct paths to neuroticism, sleep quality, and SWB. This initial GSEM was identified, limiting model fit evaluation. To address this, we used a stepwise model selection approach, sequentially removing covariates with non-significant pathways (*P* > 0.05) on the basis of likelihood ratio tests, the Akaike information criterion (AIC), and the Bayesian information criterion (BIC). Simpler models were retained if the likelihood ratio test was non-significant (*P* > 0.05) and if the AIC/BIC values decreased. The final GSEM included the following paths: (1) SWB: sleep quality, neuroticism, location, and SES; (2) sleep quality: neuroticism and age; and (3) neuroticism: age and SES. The final SEM included the following: (1) SWB: mindfulness, neuroticism, and SES; (2) mindfulness: neuroticism, sex, and age; and (3) neuroticism: age and SES. Model fit indices were evaluated for the GSEM and SEM. To estimate direct and indirect effects, we used a bootstrap approach (1000 random samples) and reported 95% bias-corrected confidence intervals.

All analyses were performed via STATA 16, with regression, gsem, and bootstrap commands for key analysis. To account for multiple hypothesis testing across the three key moderator/mediator variables, we applied a Bonferroni correction, adjusting the significance threshold to 0.017 (i.e., 0.05/3). All the statistical tests were two-tailed, and significance was set at the corrected *P* value unless otherwise noted.

### Ethical consideration

The research team obtained Institutional Review Board approval from both the University of Pennsylvania and the ethical committee for research at Jintan Hospital in China. After parental permission and adolescents’ assent forms were in place, participants received instructions from research coordinators and completed the questionnaires in their classrooms.

## Results

### Sample characteristics

Among the 543 participants who had complete data on sleep, neuroticism, and SWB, the average age at data collection was 12.98 ± 0.88 years, and 49% (*n* = 266) were females. Most of them lived in suburbs (43.28%) or urban areas (42.36%). Thirty-six percent of the participants reported poor sleep (PSQI > 5). Midsleep times shifted 1.56 hours later on weekends (3:30 a.m.) than on weekdays (1:44 a.m.), with an average mid-sleep time (corrected for sleep debt) of 2:47 a.m. Table 1 shows sample characteristics.

**Table 1:** Descriptive statistics of sample characteristics (*n* = 543)

Variables	Values
Gender, *n* (%)	
Male	277 (51.01)
Female	266 (48.99)
Region, *n* (%)	
Rural	78 (14.36)
Suburb	235 (43.28)
Urban	230 (42.36)
Age (*y*), mean ± SD	12.98 ± 0.88
SES *Z* score, mean ± SD	0.91 ± 1.18

*SES* socioeconomic status, *SD* standard deviation

### Bivariate analysis results

At the bivariate level (Table 2), poor sleepers had significantly higher neuroticism scores [*t* (541) = − 3.91, Cohen’s *d* = 0.35, *P* < 0.001] and lower SWB scores [*t* (541) = 7.10, Cohen’s *d* = 0.63, *P* < 0.001]. The average scores for neuroticism [*F* (2, 540) = 0.65, *P* = 0.52] and SWB [*F* (2, 540) = 0.695, *P* = 0.39] were lower in those with the late chronotype; however, the differences were not statistically significant. As shown in Table 3, neuroticism (*r* = − 0.22, *P* < 0.001) was negatively correlated with SWB, and trait mindfulness (*r* = 0.42, *P* < 0.001) was positively correlated with SWB. There was a negative but weak correlation between neuroticism and trait mindfulness (*r* = − 0.13, *P* = 0.03).

**Table 2:** Subjective well-being and neuroticism by sleep status (*n* = 543)

Variables	Number (%)	Neuroticism^a^	SWB^a^
Sleep quality			
Healthy sleep	345 (63.54)	2.29 ± 0.49	129.05 ± 18.24
Poor sleep	198 (36.64)	2.60 ± 0.60^†^	117.42 ± 18.55^‡^
Chronotype^b^			
Early	107 (20.19)	2.33 ± 0.97	124.33 ± 20.57
Moderate	375 (70.75)	2.43 ± 0.88	125.71 ± 18.93
Late	48 (9.06)	2.32 ± 0.88	121.88 ± 18.93
Total	543 (100)	2.41 ± 0.90	125.08 ± 19.18

*SWB* subjective well-being. ^a^Values are presented as mean ± standard deviation; ^b^chronotype has missing data. ^*^P < 0.05, ^†^*P* < 0.01, ^‡^*P* < 0.001

**Table 3:** Pearson correlations among SWB, neuroticism, and trait mindfulness (*n* = 188)

Variables	Mean ± SD	SWB	Mindfulness
SWB	125.08 ± 19.18		
Mindfulness	77.48 ± 7.71	0.42^‡^	
Neuroticism	2.46 ± 0.84	−0.22^‡^	−0.13^*^

*SWB* subjective well-being, *SD* standard deviation. ^*^P < 0.05, ^†^*P* < 0.01, ^‡^*P* < 0.001

### Multivariable analyses

Table 4 shows the results of linear regression with and without interaction terms. The associations of SWB with neuroticism (*b* = − 5.07, *P* < 0.001) and trait mindfulness (*b* = 1.06, *P* < 0.001) remained significant in the regression model, controlling for covariates. Compared with healthy sleepers, individuals with poor sleep quality (*b* = − 9.90, *P* < 0.001) were more likely to have lower SWB. Compared with the late chronotype, the moderate chronotype (*b* = 5.91, *P* = 0.04) was associated with better SWB in adolescents. The interaction terms between sleep quality and neuroticism; chronotype and neuroticism; and trait mindfulness and neuroticism were not significant (all *P* > 0.017). Among the covariates, better SES was consistently associated with greater SWB in the models (Table 4, all *P* < 0.017). There were no significant associations of gender, age, or residence districts with SWB.

**Table 4:** Linear regression models on subjective well-being [*β*(SE)]

Variables	Model 1	Model 2	Model 3	Model 4	Model 5	Model 6	Model 7
Male	2.23 (1.60)	1.84 (1.55)	1.84 (1.54)	1.84 (1.62)	1.81 (1.62)	− 2.47 (2.43)	− 2.13 (2.43)
Age	− 0.19 (0.93)	0.19 (0.90)	0.23 (0.90)	− 0.79 (0.95)	− 0.77 (0.95)	− 0.05 (2.10)	0.42 (2.11)
SES	2.27 (0.70)^†^	2.03 (0.67)^†^	1.98 (0.67)^†^	2.43 (0.70)^†^	2.37 (0.70)^*^	2.20 (1.05)^*^	2.22 (1.05)^*^
Residence							
Suburb	− 0.55 (2.24)	− 0.96 (2.35)	− 0.87 (2.35)	− 0.99 (2.47)	− 1.20 (2.47)	− 0.05 (2.10)	1.78 (3.84)
Urban	− 3.85 (2.46)	− 4.13 (2.38)	− 3.95 (2.38)	− 4.39 (2.51)	− 4.60 (2.52)	2.20 (1.05)	0.84 (3.62)
Neuroticism	− 5.07 (0.90)^‡^	− 4.26 (0.88)^‡^	− 5.38 (1.07)^‡^	− 5.04 (0.91)^‡^	− 5.79 (3.05)	− 1.24 (1.42)	26.44 (15.79)
Poor sleep		− 9.90 (1.62)^‡^	− 18.12 (4.90)^†^				
Neuroticism × poor sleep			3.28 (1.85)				
Chronotype							
Early				4.22 (3.25)	7.27 (8.86)		
Moderate				5.91 (2.87)^*^	2.23 (8.05)		
Neuroticism × chronotype							
Early					− 1.33 (3.56)		
Moderate					1.54 (3.23)		
Mindfulness						1.06 (0.16)^‡^	1.86 (0.48)^‡^
Neuroticism × mindfulness							− 0.36 (0.20)
*R*^2^	0.08	0.15	0.14	0.08	0.08	0.21	0.19

Sample size in models 1–5: *n* = 543, model 6 and 7: *n* = 188. Reference levels: residence: rural; poor sleep: healthy sleep, chronotype: late. *R*^2^ variance explained by the model. *SES* socioeconomic status, *SE* standard error. ^*^P < 0.05, ^†^*P* < 0.01, ^‡^*P* < 0.001

**Fig. 1:**
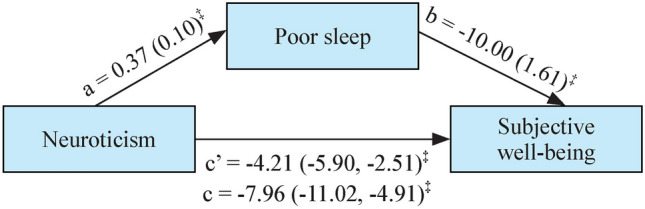
Direct and indirect effects of neuroticism on subjective well-being [*β*(SE)]: poor sleep as a mediator (*n* = 543). a: Direct effect of neuroticism on poor sleep quality; b: direct effect of poor sleep on subjective well-being; c’: direct effect of neuroticism on subjective well-being; c: total effect of neuroticism on subjective well-being. *SE* standard error. ^‡^*P* < 0.001; biased corrected 95% confidence interval from 1000 bootstrap samples

**Fig. 2:**
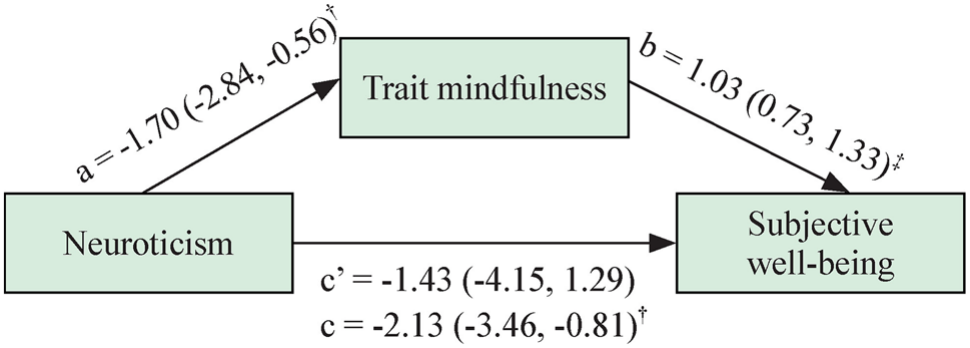
Direct and indirect effects of neuroticism scores on subjective well-being [*β*(SE)]: mindfulness as a mediator (*n* = 188). a: Direct effect of neuroticism on trait mindfulness; b: direct effect of trait mindfulness on subjective well-being; c’: direct effect of neuroticism on subjective well-being; c: total effect of neuroticism on subjective well-being. *SE* standard error. ^†^*P* < 0.01, ^‡^*P* < 0.001; biased corrected 95% confidence interval from 1000 bootstrap samples

Both sleep quality and trait mindfulness significantly mediated the association between neuroticism and SWB (*P* < 0.05). As shown in Fig. 1, among those with complete data on neuroticism (*n* = 543), increased neuroticism was associated with an increased likelihood of poor sleep quality (*β* = 0.37, *P* < 0.001), which in turn was associated with decreased SWB (*β* = − 10.00, *P* < 0.001). Bootstrapping estimation suggested that poor sleep quality accounted for 47.23% (*β* = − 3.76, *P* = 0.002) of the total effect of neuroticism on SWB (*β* = − 7.97, *P* < 0.001). The AIC and BIC for the GESM were 6790.59 and 6846.45, respectively. Among the subsample with mindfulness data, trait mindfulness fully mediated the association between neuroticism and SWB (*β* = − 2.13, *P* = 0.002), as the direct pathway was not significant (*β* = − 1.43, *P* = 0.22) (Fig. 2). The model demonstrated good fit, as indicated by a non-significant likelihood ratio test comparing the SEM to the saturated model (*χ*^2^ = 4.34, *P* = 0.362), along with favorable fit indices: root-mean-square error of approximation = 0.02, AIC = 5779.05, BIC = 5826.82, comparative fit index = 0.99, and Tucker‒Lewis index = 0.99. There were no significant pathways associated with chronotypes according to the mediation analyses (*P* > 0.017).

## Discussion

This study represents one of the first to explore the roles of sleep quality, chronotype, and mindfulness in the association between neuroticism and SWB in early adolescents. Early adolescents with higher levels of neuroticism, poor sleep quality, and a later chronotype tended to experience worse SWB. In contrast, those with greater trait mindfulness were associated with better SWB. Poor sleep and mindfulness, but not chronotype, mediated the association between neuroticism and SWB. There were no moderating effects of sleep quality, chronotype, or mindfulness on the relationship between neuroticism and SWB. Our findings underscore the importance of sleep health and mindfulness practices for promoting adolescent health, particularly among those with high neuroticism.

Our study extends previous work by examining the mediating role of sleep or trait mindfulness in the relationship between neuroticism and SWB among early adolescents. Consistent with existing evidence, high neuroticism was a risk factor for compromised SWB, and this relationship was mediated by sleep quality. A meta-analysis has indicated that neuroticism, including positive affect and life satisfaction, is the strongest personality correlate of SWB across adolescent and adult samples [24]. High neuroticism has been linked to heightened emotional reactivity, partly due to increased activation in the hippocampal–parahippocampal complex related to fear learning and reduced activation in brain regions related to the anticipation of aversive stimuli [61]. While a few studies have tested sleep as a mediator between neuroticism and SWB, prior research has consistently shown that neuroticism is the strongest predictor of poor sleep quality among the Big Five personality traits [30]. Adolescents high in neuroticism often exhibit greater cortisol reactivity and pre-sleep arousal after stress, leading to disrupted sleep patterns [62]. Poor sleep quality, in turn, affects neuronal networks and brain regions (i.e., the prefrontal cortex and amygdala) involved in emotional regulation and stress coping, which may compromise SWB [63, 64].

In our sample, neuroticism was not associated with chronotype and therefore did not serve as a mediator in this study. This finding contrasts with prior meta-analyses linking higher neuroticism to a lower tendency toward morning circadian preference [26]. One possible explanation for this discrepancy lies in differences in morningness and eveningness questionnaires [26]. In support of our findings, a large-scale study of Estonian adults that used the same measure revealed that neuroticism was the only personality trait that was not significantly correlated with chronotype [65]. Despite the inconsistent findings on neuroticism, our study suggested that a later chronotype was independently associated with poorer SWB in early adolescents, which aligns with previous research [8–10]. Adolescents with later chronotypes often experience circadian misalignment between biological and social schedules, short sleep duration, and poor sleep quality, all of which contribute to worse SWB [66]. More research is needed to examine how neuroticism, chronotype, and SWB are interrelated across developmental stages and cultural contexts.

Our findings highlight the important role of mindfulness in the link between neuroticism and SWB. As a positive personality, trait mindfulness was positively associated with SWB. The capacity to stay present and attentive through mindfulness may help buffer against stress and negative emotions [16, 17], thereby promoting well-being during the developmental and social challenges of adolescence. However, adolescents high in neuroticism tended to report lower mindfulness, which in turn predicted poorer SWB. This mediating role of mindfulness aligns with findings from adult populations [18]. Notably, a study on adults suggested that mindfulness was only a significant mediator of high levels of neuroticism [18]. These results underscore the potential benefit of mindfulness-based interventions to increase well-being among adolescents, particularly those with greater neuroticism.

Prior studies have also suggested healthy sleep, earlier chronotype or high mindfulness as potential protective factors against negative personality traits, such as neuroticism, among young adults and older adults [32–34]. In contrast, we found that these factors did not moderate the association between neuroticism and SWB in early adolescents. Several explanations may account for this discrepancy. First, prior work has examined outcomes, such as executive function and depressive symptoms [32–34], whereas we focused on SWB, which may be influenced by different regulatory mechanisms. Second, whereas prior studies focused primarily on adult or older adult populations, our findings were based on early adolescents, a developmental period characterized by unique vulnerabilities. Adolescents are in a period of ongoing brain maturation, especially in prefrontal regions critical for emotion regulation and executive control, which may limit the buffering effects of sleep, chronotype, and mindfulness. Third, measurement constraints (e.g., brief self-report scales) may have reduced sensitivity in detecting subtle interactive effects. It is also possible that in early adolescence, the relationship between neuroticism and well-being operates more through mediating mechanisms rather than moderation. Future research should examine these pathways longitudinally and explore contextual and developmental influences that may shape these associations across age groups.

Several limitations should be noted in this study. First, the absence of temporal data limits our ability to make causal inferences. Neuroticism, sleep, mindfulness, and SWB may influence each other in a bidirectional manner. Future longitudinal studies or clinical trials are warranted to establish causal relationships. Second, subjective measures of sleep quality and chronotype are subject to recall biases. Incorporating objective sleep measures (e.g., actigraphy) will strengthen the study’s validity. Third, mindfulness analyses were limited by incomplete data availability. The sample characteristics of the subsample with complete mindfulness data differ from those of the full sample included in this study. While neuroticism showed a significant direct pathway to SWB in the full sample, this relationship was non-significant in the mindfulness subsample, potentially indicating full mediation. However, this non-significance may reflect reduced statistical power rather than true full mediation effects. Fourth, the sample size reduction from the initial sample size of the Jintan Child Cohort study raises concerns about attrition bias. Despite multi-stage sampling, the final analytic sample of 543 participants may not fully represent all adolescents in this county-level city, potentially limiting generalizability. Fifth, trait mindfulness data were collected approximately one year after the other variables (2013–2014), primarily among participants from the middle and lower preschool cohorts who had completed assessments of neuroticism, sleep, and SWB in 2012–2013. While trait mindfulness is considered relatively stable, this temporal gap may influence interpretation. Future research using larger, nationally representative samples and both cross-sectional and longitudinal designs is needed to replicate and extend these findings. Sixth, the BFI-10's neuroticism subscale contains only two items, which constrains internal consistency estimates. Future research utilizing more comprehensive neuroticism measures (e.g., the BFI-44) is needed to achieve more robust estimates. Finally, while our sample focused on early adolescents in China, future work should explore whether these findings can be generalized across different adolescent stages and cultural contexts.

Despite its study limitations, this study has significant public health implications. Personality traits such as neuroticism are relatively stable across the lifespan; however, adolescents—particularly adolescent girls—tend to report higher levels of neuroticism than children and adults do [20]. This makes adolescence a sensitive developmental period during which targeted interventions could have a substantial impact. Given that neuroticism independently predicts poorer sleep quality and that sleep disturbances can further compromise SWB, pediatric healthcare providers should prioritize sleep assessment and intervention, particularly among adolescents exhibiting strong neuroticism tendencies. In our sample, approximately 36% of our participants were classified as poor sleepers, and 10% of them had sleep midpoints later than 4 a.m. School- or clinic-based programs incorporating age-appropriate sleep interventions, such as sleep hygiene education, cognitive‒behavioral therapy for insomnia adapted for adolescents, and circadian rhythm regulation strategies, may be crucial in disrupting the cycle of poor sleep and diminished well-being. Similarly, mindfulness training—delivered through brief, developmentally tailored programs—may enhance adolescents’ capacity for emotional regulation and reduce the impact of neurotic tendencies on daily functioning.

In conclusion, both poor sleep and low mindfulness mediated the association between neuroticism and SWB, while no significant interaction effects were observed. Our findings underscore the need for early identification and support of adolescents high in neuroticism, who may be at elevated risk for multiple adverse outcomes, including poor sleep, reduced well-being, and potentially worsening mental health difficulties. Integrating sleep assessment and sleep interventions, as well as mindfulness practices, into routine care could enable more personalized and effective prevention strategies during this formative developmental period. Future research should test the temporal associations in other populations and examine targeted interventions that may mitigate the adverse effects of neuroticism on well-being.

## Data Availability

The datasets generated during and/or analyzed during the current study are not publicly available due to data sharing policy from the funding agency and universities but are available from the corresponding author on reasonable request.

## References

[1] Chen T, Wu Z, Shen Z, Zhang J, Shen X, Li S. Sleep duration in Chinese adolescents: biological, environmental, and behavioral predictors. Sleep Med. 2014;15:1345–53. 25277663 10.1016/j.sleep.2014.05.018

[2] Gradisar M, Gardner G, Dohnt H. Recent worldwide sleep patterns and problems during adolescence: a review and meta-analysis of age, region, and sleep. Sleep Med. 2011;12:110–8. 21257344 10.1016/j.sleep.2010.11.008

[3] Short MA, Gradisar M, Lack LC, Wright HR, Dewald JF, Wolfson AR, et al. A cross-cultural comparison of sleep duration between US and Australian adolescents: the effect of school start time, parent-set bedtimes, and extracurricular load. Health Educ Behav. 2013;40:323–30. 22984209 10.1177/1090198112451266 PMC4232364

[4] Olds T, Blunden S, Petkov J, Forchino F. The relationships between sex, age, geography and time in bed in adolescents: a meta-analysis of data from 23 countries. Sleep Med Rev. 2010;14:371–8. 20207558 10.1016/j.smrv.2009.12.002

[5] Ramar K, Malhotra RK, Carden KA, Martin JL, Abbasi-Feinberg F, Aurora RN, et al. Sleep is essential to health: an American academy of sleep medicine position statement. J Clin Sleep Med. 2021;17:2115–9. 34170250 10.5664/jcsm.9476 PMC8494094

[6] Kocevska D, Lysen TS, Dotinga A, Koopman-Verhoeff ME, Luijk MPCM, Antypa N, et al. Sleep characteristics across the lifespan in 1.1 million people from the Netherlands, United Kingdom and United States: a systematic review and meta-analysis. Nat Hum Behav. 2021;5:113–22. 33199855 10.1038/s41562-020-00965-x

[7] Roenneberg T. Having trouble typing? What on earth is chronotype? J Biol Rhythms. 2015;30:487–91. 26446872 10.1177/0748730415603835

[8] Otsuka Y, Kaneita Y, Itani O, Jike M, Osaki Y, Higuchi S, et al. The relationship between subjective happiness and sleep problems in Japanese adolescents. Sleep Med. 2020;69:120–6. 32062038 10.1016/j.sleep.2020.01.008

[9] Vermeulen MC, van der Heijden KB, Kocevska D, Treur JL, Huppertz C, van Beijsterveldt CE, et al. Associations of sleep with psychological problems and well-being in adolescence: causality or common genetic predispositions? J Child Psychol Psychiatr. 2021;62:28–39. 10.1111/jcpp.13238 PMC7818180 32396669

[10] An Y, Ji X, Zhou L, Liu J. Sleep and subjective well-being among Chinese adolescents: resilience as a mediator. Asian J Soc Health Behav. 2023;6:112–8.

[11] Steinmayr R, Wirthwein L, Modler L, Barry MM. Development of subjective well-being in adolescence. Int J Environ Res Public Health. 2019;16:3690. 31575056 10.3390/ijerph16193690 PMC6801746

[12] Chervonsky E, Hunt C. Emotion regulation, mental health, and social wellbeing in a young adolescent sample: a concurrent and longitudinal investigation. Emotion. 2019;19:270. 29697988 10.1037/emo0000432

[13] Datu JAD, King RB. Subjective well-being is reciprocally associated with academic engagement: a two-wave longitudinal study. J Sch Psychol. 2018;69:100–10. 30558746 10.1016/j.jsp.2018.05.007

[14] Arslan G, Renshaw TL. Student subjective wellbeing as a predictor of adolescent problem behaviors: a comparison of first-order and second-order factor effects. Child Indic Res. 2018;11:507–21.

[15] Carskadon MA, Acebo C, Jenni OG. Regulation of adolescent sleep: implications for behavior. Ann N Y Acad Sci. 2004;1021:276–91. 15251897 10.1196/annals.1308.032

[16] Hanley AW, Garland EL. The mindful personality: a meta-analysis from a cybernetic perspective. Mindfulness. 2017;8:1456–70. 29479377 10.1007/s12671-017-0736-8 PMC5822739

[17] Giluk TL. Mindfulness, big five personality, and affect: a meta-analysis. Pers Individ Differ. 2009;47:805–11.

[18] Wenzel M, Von Versen C, Hirschmüller S, Kubiak T. Curb your neuroticism–mindfulness mediates the link between neuroticism and subjective well-being. Pers Individ Differ. 2015;80:68–75.

[19] Costa JPT, McCrae RR. Neuroticism, somatic complaints, and disease: is the bark worse than the bite? J Pers. 1987;55:299–316. 3612472 10.1111/j.1467-6494.1987.tb00438.x

[20] Soto CJ, John OP, Gosling SD, Potter J. Age differences in personality traits from 10 to 65: big five domains and facets in a large cross-sectional sample. J Pers Soc Psychol. 2011;100:330–48. 21171787 10.1037/a0021717

[21] Haas BW, Omura K, Constable RT, Canli T. Emotional conflict and neuroticism: personality-dependent activation in the amygdala and subgenual anterior cingulate. Behav Neurosci. 2007;121:249–56. 17469914 10.1037/0735-7044.121.2.249

[22] Hisler GC, Krizan Z, DeHart T, Wright AG. Neuroticism as the intensity, reactivity, and variability in day-to-day affect. J Res Pers. 2020;87:103964.

[23] Singh P. Emotion regulation difficulties mediate the relationship between neuroticism and health-risk behaviours in adolescents. J Psychol. 2022;156:48–67. 35015628 10.1080/00223980.2021.2006124

[24] Anglim J, Horwood S, Smillie LD, Marrero RJ, Wood JK. Predicting psychological and subjective well-being from personality: a meta-analysis. Psychol Bull. 2020;146:279–323. 31944795 10.1037/bul0000226

[25] Okbay A, Baselmans BM, De Neve JE, Turley P, Nivard MG, Fontana MA, et al. Genetic variants associated with subjective well-being, depressive symptoms, and neuroticism identified through genome-wide analyses. Nat Genet. 2016;48:624–33. 27089181 10.1038/ng.3552 PMC4884152

[26] Lipnevich AA, Credè M, Hahn E, Spinath FM, Roberts RD, Preckel F. How distinctive are morningness and eveningness from the Big Five factors of personality? A meta-analytic investigation. J Pers Soc Psychol. 2017;112:491–509. 27977220 10.1037/pspp0000099

[27] Duggan KA, Friedman HS, McDevitt EA, Mednick SC. Personality and healthy sleep: the importance of conscientiousness and neuroticism. PLoS ONE. 2014;9:e90628. 24651274 10.1371/journal.pone.0090628 PMC3961248

[28] Slavish DC, Sliwinski MJ, Smyth JM, Almeida DM, Lipton RB, Katz MJ, et al. Neuroticism, rumination, negative affect, and sleep: examining between-and within-person associations. Pers Individ Dif. 2018;123:217–22. 29610545 10.1016/j.paid.2017.11.023 PMC5877474

[29] Zamani E, Akbari M, Mohammadkhani S, Riskind JH, Drake CL, Palagini L. The relationship of neuroticism with sleep quality: the mediating role of emotional, cognitive and metacognitive factors. Behav Sleep Med. 2022;20:74–89. 33618569 10.1080/15402002.2021.1888730

[30] Cellini N, Duggan KA, Sarlo M. Perceived sleep quality: the interplay of neuroticism, affect, and hyperarousal. Sleep Health. 2017;3:184–9. 28526256 10.1016/j.sleh.2017.03.001

[31] Dong XX, Huang Y, Miao YF, Hu HH, Pan CW, Zhang T, et al. Personality and health-related quality of life of older Chinese adults: cross-sectional study and moderated mediation model analysis. JMIR Public Health Surveill. 2024;10:e57437. 39267352 10.2196/57437 PMC11412092

[32] Kim BR, Lee R, Kim N, Jeong JH, Kim GH. The moderating role of sleep quality on the association between neuroticism and frontal executive function in older adults. Behav Sleep Med. 2022;20:50–62. 33522299 10.1080/15402002.2021.1879809

[33] Gorgol J, Waleriańczyk W, Stolarski M. The moderating role of personality traits in the relationship between chronotype and depressive symptoms. Chronobiol Int. 2022;39:106–16. 34612109 10.1080/07420528.2021.1979995

[34] Feltman R, Robinson MD, Ode S. Mindfulness as a moderator of neuroticism–outcome relations: a self-regulation perspective. J Res Pers. 2009;43:953–61.

[35] González-Carrasco M, Casas F, Malo S, Viñas F, Dinisman T. Changes with age in subjective well-being through the adolescent years: differences by gender. J Happiness Stud. 2017;18:63–88.

[36] Damian RI, Spengler M, Sutu A, Roberts BW. Sixteen going on sixty-six: a longitudinal study of personality stability and change across 50 years. J Pers Soc Psychol. 2019;117:674–95. 30113194 10.1037/pspp0000210

[37] Anusic I, Schimmack U. Stability and change of personality traits, self-esteem, and well-being: introducing the meta-analytic stability and change model of retest correlations. J Pers Soc Psychol. 2016;110:766–81. 26619304 10.1037/pspp0000066

[38] Brown TA, Barlow DH. A proposal for a dimensional classification system based on the shared features of the DSM-IV anxiety and mood disorders: implications for assessment and treatment. Psychol Assess. 2009;21:256–71. 19719339 10.1037/a0016608 PMC2845450

[39] Monroe SM, Simons AD. Diathesis-stress theories in the context of life stress research: implications for the depressive disorders. Psychol Bull. 1991;110:406–25. 1758917 10.1037/0033-2909.110.3.406

[40] Buysse DJ, Reynolds IIICF, Monk TH, Berman SR, Kupfer DJ. The Pittsburgh sleep quality index: a new instrument for psychiatric practice and research. Psychiatry Res. 1989;28:193–213. 2748771 10.1016/0165-1781(89)90047-4

[41] Buysse DJ. Sleep health: can we define it? Does it matter? Sleep. 2014;37:9–17. 24470692 10.5665/sleep.3298 PMC3902880

[42] Dong L, Martinez AJ, Buysse DJ, Harvey AG. A composite measure of sleep health predicts concurrent mental and physical health outcomes in adolescents prone to eveningness. Sleep Health. 2019;5:166–74. 30928117 10.1016/j.sleh.2018.11.009 PMC6452900

[43] Liu J, Cao S, Chen Z, Raine A, Hanlon A, Ai Y, et al. Cohort profile update: the China Jintan child cohort study. Int J Epidemiol. 2015;44:1548. 26323725 10.1093/ije/dyv119 PMC4707195

[44] Liu J, McCauley LA, Zhao Y, Zhang H, Pinto-Martin J, Jintan Cohort Study Group. Cohort profile: the China Jintan child cohort study. Int J Epidemiol. 2010;39:668–74. 19433517 10.1093/ije/dyp205 PMC2912482

[45] Hills P, Argyle M. The Oxford happiness questionnaire: a compact scale for the measurement of psychological well-being. Pers Individ Differ. 2002;33:1073–82.

[46] Li Y, Chen Y. Dimensional structure of Oxford happiness questionnaire (revision) and verification of its reliability and validity. Health Med Res Pract. 2013;10:34–41.

[47] John B, Bellipady SS, Bhat SU. Sleep promotion program for improving sleep behaviors in adolescents: a randomized controlled pilot study. Scientifica. 2016;2016:8013431. 27088040 10.1155/2016/8013431 PMC4818821

[48] Hirshkowitz M, Whiton K, Albert SM, Alessi C, Bruni O, DonCarlos L, et al. National sleep foundation’s sleep time duration recommendations: methodology and results summary. Sleep Health. 2015;1:40–3. 29073412 10.1016/j.sleh.2014.12.010

[49] Zhou HQ, Shi WB, Wang XF, Yao M, Cheng GY, Chen PY, et al. An epidemiological study of sleep quality in adolescents in South China: a school-based study. Child Care Health Dev. 2012;38:581–7. 21831260 10.1111/j.1365-2214.2011.01300.x

[50] Roenneberg T, Kuehnle T, Juda M, Kantermann T, Allebrandt K, Gordijn M, et al. Epidemiology of the human circadian clock. Sleep Med Rev. 2007;11:429–38. 17936039 10.1016/j.smrv.2007.07.005

[51] Carciofo R, Yang J, Song N, Du F, Zhang K. Psychometric evaluation of Chinese-language 44-item and 10-item big five personality inventories, including correlations with chronotype, mindfulness and mind wandering. PLoS ONE. 2016;11:e0149963. 26918618 10.1371/journal.pone.0149963 PMC4769279

[52] Rammstedt B, John OP. Measuring personality in one minute or less: a 10-item short version of the big five inventory in English and German. J Res Pers. 2007;41:203–12.

[53] Bohlmeijer E, Ten Klooster PM, Fledderus M, Veehof M, Baer R. Psychometric properties of the five facet mindfulness questionnaire in depressed adults and development of a short form. Assessment. 2011;18:308–20. 21586480 10.1177/1073191111408231

[54] Baer RA, Smith GT, Hopkins J, Krietemeyer J, Toney L. Using self-report assessment methods to explore facets of mindfulness. Assessment. 2006;13:27–45. 16443717 10.1177/1073191105283504

[55] Brady B, Kneebone II, Bailey PE. Validation of the five facet mindfulness questionnaire among community-dwelling older adults. Mindfulness. 2019;10:529–36.

[56] Ye T, Cui N, Yang W, Liu J. Evaluation of the factor structure of the adolescent stress questionnaire in Chinese adolescents. Psychol Rep. 2019;122:2366–95. 30189799 10.1177/0033294118792686 PMC6669116

[57] Hou J, Wong SY, Lo HH, Mak WW, Ma HS. Validation of a Chinese version of the five facet mindfulness questionnaire in Hong Kong and development of a short form. Assessment. 2014;21:363–71. 23596271 10.1177/1073191113485121

[58] Straus MA. Prevalence of violence against dating partners by male and female university students worldwide. Violence Against Women. 2004;10:790–811.

[59] Jeriček Klanšček H, Zager Kocjan G, Roškar S. Predictors of life satisfaction in Slovenian 15-year-old adolescents. Eur J Public Health. 2018;28:cky214.

[60] Steinvoord K, Junge A. Does an association exist between socio-economic status and subjective physical, mental and social well-being among early adolescents? Int J Adolesc Med Health. 2022;34:20190090. 10.1515/ijamh-2019-0090 31586965

[61] Servaas MN, Van Der Velde J, Costafreda SG, Horton P, Ormel J, Riese H, et al. Neuroticism and the brain: a quantitative meta-analysis of neuroimaging studies investigating emotion processing. Neurosci Biobehav Rev. 2013;37:1518–29. 23685122 10.1016/j.neubiorev.2013.05.005

[62] Slavish DC. The role of neuroticism in daily experiences, affect, and nightly sleep quality. State College: The Pennsylvania State University; 2017.

[63] Maier SF, Watkins LR. Role of the medial prefrontal cortex in coping and resilience. Brain Res. 2010;1355:52–60. 20727864 10.1016/j.brainres.2010.08.039 PMC2967290

[64] Yoo SS, Gujar N, Hu P, Jolesz FA, Walker MP. The human emotional brain without sleep—a prefrontal amygdala disconnect. Curr Biol. 2007;17:R877–8. 17956744 10.1016/j.cub.2007.08.007

[65] Lenneis A, Vainik U, Teder-Laving M, Ausmees L, Lemola S, Allik J, et al. Personality traits relate to chronotype at both the phenotypic and genetic level. J Pers. 2021;89:1206–22. 33998684 10.1111/jopy.12645

[66] Lan A, Stukalin Y, Einat H. Sleep quality, but not personality traits, mediates the relationship between chronotype and life satisfaction: a study in young adults. Clocks Sleep. 2024;6:312–21. 39189189 10.3390/clockssleep6030022 PMC11348206

